# Dissociation of Tau Deposits and Brain Atrophy in Early Alzheimer’s Disease: A Combined Positron Emission Tomography/Magnetic Resonance Imaging Study

**DOI:** 10.3389/fnagi.2018.00223

**Published:** 2018-07-18

**Authors:** Yoko Shigemoto, Daichi Sone, Etsuko Imabayashi, Norihide Maikusa, Nobuyuki Okamura, Shozo Furumoto, Yukitsuka Kudo, Masayo Ogawa, Harumasa Takano, Yuma Yokoi, Masuhiro Sakata, Tadashi Tsukamoto, Koichi Kato, Noriko Sato, Hiroshi Matsuda

**Affiliations:** ^1^Integrative Brain Imaging Center, National Center of Neurology and Psychiatry, Tokyo, Japan; ^2^Department of Radiology, National Center of Neurology and Psychiatry, Tokyo, Japan; ^3^Department of Psychiatry, National Center of Neurology and Psychiatry, Tokyo, Japan; ^4^Division of Pharmacology, Faculty of Medicine, Tohoku Medical and Pharmaceutical University, Sendai, Japan; ^5^Division of Neuro-imaging, Institute of Development, Aging and Cancer, Tohoku University, Sendai, Japan; ^6^Division of Radiopharmaceutical Chemistry, Cyclotron and Radioisotope Center, Tohoku University, Sendai, Japan; ^7^Department of Neurology, National Center of Neurology and Psychiatry, Tokyo, Japan

**Keywords:** Alzheimer’s disease, tau, positron emission tomography, magnetic resonance imaging, partial volume correction, brain atrophy

## Abstract

The recent advent of tau-specific positron emission tomography (PET) has enabled *in vivo* assessment of tau pathology in Alzheimer’s disease (AD). However, because PET scanners have limited spatial resolution, the measured signals of small brain structures or atrophied areas are underestimated by partial volume effects (PVEs). The aim of this study was to determine whether partial volume correction (PVC) improves the precision of measures of tau deposits in early AD. We investigated tau deposits in 18 patients with amyloid-positive early AD and in 36 amyloid-negative healthy controls using ^18^F-THK5351 PET. For PVC, we applied the SPM toolbox PETPVE12. The PET images were then spatially normalized and subjected to voxel-based group analysis using SPM12 for comparison between the early AD patients and healthy controls. We also compared these two groups in terms of brain atrophy using voxel-based morphometry of MRI. We found widespread neocortical tracer retention predominantly in the posterior cingulate and precuneus areas, but also in the inferior temporal lobes, inferior parietal lobes, frontal lobes, and occipital lobes in the AD patients compared with the controls. The pattern of tracer retention was similar between before and after PVC, suggesting that PVC had little effect on the precision of tau load measures. Gray matter atrophy was detected in the medial/lateral temporal lobes and basal frontal lobes in the AD patients. Interestingly, only a few associations were found between atrophy and tau deposits, even after PVC. In conclusion, PVC did not significantly affect ^18^F-THK5351 PET measures of tau deposits. This discrepancy between tau deposits and atrophy suggests that tau load precedes atrophy.

## Introduction

Alzheimer’s disease (AD), the most common form of progressive degenerative dementia, is characterized by cognitive deterioration and behavioral impairment. One of the neuropathological hallmarks of AD is the aggregation of hyperphosphorylated tau protein into intracellular neurofibrillary tangles (NFTs) (Braak and Braak, 1991). Tau pathology correlates with cognitive functions better than amyloid-β (Nelson et al., 2012; Murray et al., 2015). The recent advent of tau-specific positron emission tomography (PET) has enabled *in vivo* assessment of tau pathology in AD, which will aid in early detection, disease staging, and treatment development (Holtzman et al., 2016).

Because PET scanners have limited spatial resolution, the measured signals of small brain structures such as cortical gray matter are underestimated by partial volume effects (PVEs) (Matsuda et al., 2002; Yanase et al., 2005; Gonzalez-Escamilla et al., 2017). AD is characterized by progressive brain atrophy and thus PVEs are estimated to be even more severe in AD patients (Scheltens et al., 1992; Schroeter et al., 2009; Rullmann et al., 2016). Although some previous studies reported that PVC was effective for evaluating tau load in AD (Villemagne et al., 2014; Shidahara et al., 2017), other studies found that PVC produced only small effects (Ossenkoppele et al., 2016; Schöll et al., 2016; Maass et al., 2017). The aim of the present study was to determine whether PVC improves the precision of ^18^F-THK5351 PET measures of tau deposits in early AD.

## Materials and Methods

### Participants

In total, 54 participants – 18 patients (13 women, 5 men) with early AD and 36 cognitively normal healthy controls (20 women, 16 men) – underwent both ^18^F-THK5351 and ^11^C-PiB PET scans from June 2015 to January 2017. The average interval between ^18^F-THK5351, ^11^C-PiB PET, and MRI was 20 ± 20 days (^18^F-THK5351 to ^11^C-PiB: 12 ± 13 days; ^18^F-THK5351 to MRI: 12 ± 14 days; ^11^C-PiB to MRI: 17 ± 21 days). Diagnosis of probable AD was based on the clinical criteria outlined by the National Institute on Aging-Alzheimer’s Association (NIA-AA) (McKhann et al., 2011). All early AD patients showed visually positive ^11^C-PiB PET results. None had a history of neurological disorder causing dementia, significant cerebrovascular disease, or major systemic disease. The AD patients were 70.4 ± 7.9 years old [mean ± standard deviation (SD)], their average Mini-Mental State Examination (MMSE) score was 22.0 ± 4.5 (mean ± SD), their global Clinical Dementia Rating (CDR) ranged from 0.5 to 1.0, and CDR sum of boxes was 4.6 ± 3.0.

The cognitively normal controls showed visually negative ^11^C-PiB PET results. None had a history of neurological or psychiatric disorders and none were taking medications that affect cognition. The controls were 66.0 ± 8.6 years old, with an average MMSE score of 29.2 ± 0.9, a global CDR of 0, and CDR sum of boxes of 0.0 ± 0.3. Participants’ demographic and clinical characteristics are presented in **Table 1**.

**Table 1:** Demographic characteristics of participants.

	AD patients	CN subjects
*N*	18	36
Age	70.4 ± 7.9	66.0 ± 8.6
Gender (F/M)	13/5	20/16
MMSE	22.0 ± 4.5	29.2 ± 0.9
CDR	0.5 or 1.0	0
CDR sum of boxes	4.6 ± 3.0	0.0 ± 0.3

*AD, Alzheimer’s disease; CN, cognitively normal. Data are mean ± standard deviation; N, number of participants; F/M, female/male; MMSE, Mini-Mental Status Examination; CDR, Clinical Dementia Rating.*

All participants gave written informed consent to participate in the study, which was approved by the institutional ethics committee at the National Center of Neurology and Psychiatry.

### Image Acquisition

All participants underwent MRI scans with a 3-T MR imaging system (Verio; Siemens, Erlangen, Germany). Three-dimensional (3D) sagittal T1-weighted magnetization-prepared rapid acquisition with gradient echo (MPRAGE) images were acquired as follows: repetition time (TR)/echo time (TE), 1.900 ms/2.52 ms; flip angle (FA), 9°; in-plane resolution, 1.0 mm × 1.0 mm; 1.0 mm effective slice thickness, gapless; 300 slices; matrix, 256 × 256; field of view (FOV), 25 cm × 25 cm; acquisition time, 4 min 18 s.

Positron emission tomography scans were performed on a combined PET/CT scanner (Biograph 16; Siemens) in 3D acquisition mode. A low-dose CT scan was performed for attenuation correction before all scans. For ^18^F-THK5351 imaging, ^18^F-THK5351 at a dose of 185 MBq was injected intravenously 40 min before the PET/CT scan. For ^11^C-PiB imaging, ^11^C-PiB at a dose of 555 MBq was injected 50 min before the scan. PET/CT data were reconstructed using a combination of Fourier rebinning and ordered subset expectation maximization.

### Image Analyses

The ^18^F-THK5351 PET data were PVE-corrected using the recently developed statistical parametric mapping (SPM) toolbox PETPVE12 (Gonzalez-Escamilla et al., 2017). We used the Müller-Gärtner approach (Müller-Gärtner et al., 1992). For voxel-based analyses, both non-corrected and PVE-corrected PET images were spatially normalized using SPM version 12 (Wellcome Trust Centre for Neuroimaging, London, United Kingdom). PET scans were coregistered to the participants’ T1-weighted images and normalized with diffeomorphic anatomical registration using exponentiated Lie algebra (DARTEL). Each PET image was warped using the deformation fields derived from DARTEL registration of the coregistered T1-weighted image to the reference template. Standardized uptake value ratios (SUVR) for PET images were calculated using the mean activity in the cerebellar gray matter as the reference region. Finally, each PET image was smoothed using an 8 mm full width at half-maximum (FWHM) Gaussian kernel.

The gray matter images automatically segmented through the PVC process were also spatially normalized by DARTEL and smoothed using an 8 mm FWHM Gaussian kernel.

To investigate the direct correlation of atrophy and PVE-corrected ^18^F-THK5351 retention, 114 cerebral cortical regions of interest (ROIs) were used for calculation of both gray matter volume and ^18^F-THK5351 SUVR using Neuromorphometrics atlas^1^.

### Statistical Analyses

Differences in tau deposits between the early AD patients and healthy controls, were evaluated by a group comparison using a two-sample *t*-test in SPM12 with non-corrected and PVE-corrected ^18^F-THK5351 PET images. We also evaluated morphological group differences on gray matter images using a two-sample *t*-test in SPM12. Both statistical models included age and sex as covariates. Differences that met the following criteria were deemed significant: a height threshold of *p* < 0.001 and an extent threshold of *p* < 0.05 (corrected for multiple comparisons using the family-wise error rate).

We evaluated the correlation of atrophy and ^18^F-THK5351 retention obtained from the same ROIs using multiple regression models among AD patients and healthy controls respectively. Bonferroni and false discovery rate (FDR) correction were applied to correct multiple comparisons. Data analysis was performed using the SPSS Software, version 25.0 (SPSS, Tokyo, Japan).

Additionally, for the correlation analyses with ^18^F-THK5351 retention and the cognitive test scores (MMSE and CDR sum of boxes) within AD patients, we used a multiple regression analysis corrected for multiple comparisons in SPM12.

## Results

**FIGURE 1:**
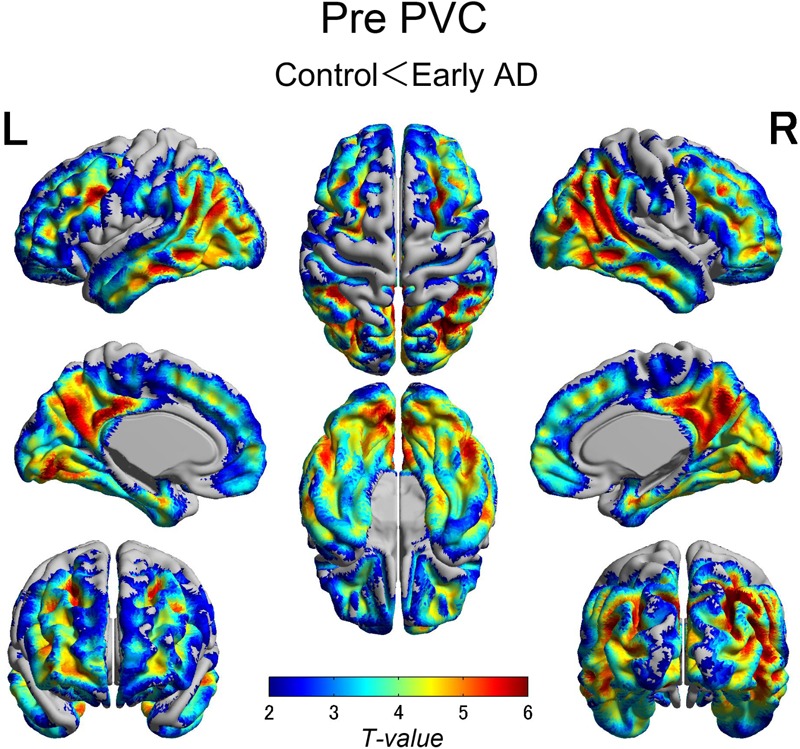
The distribution of voxel-wise group differences before PVC with ^18^F-THK5351. Widespread neocortical tracer retention was observed predominantly in the posterior cingulate/precuneus areas, as well as in the inferior temporal lobes, parieto-occipital, and frontal lobes, in the early AD patients compared with the healthy controls. AD, Alzheimer’s disease; PVC, partial volume correction.

**FIGURE 2:**
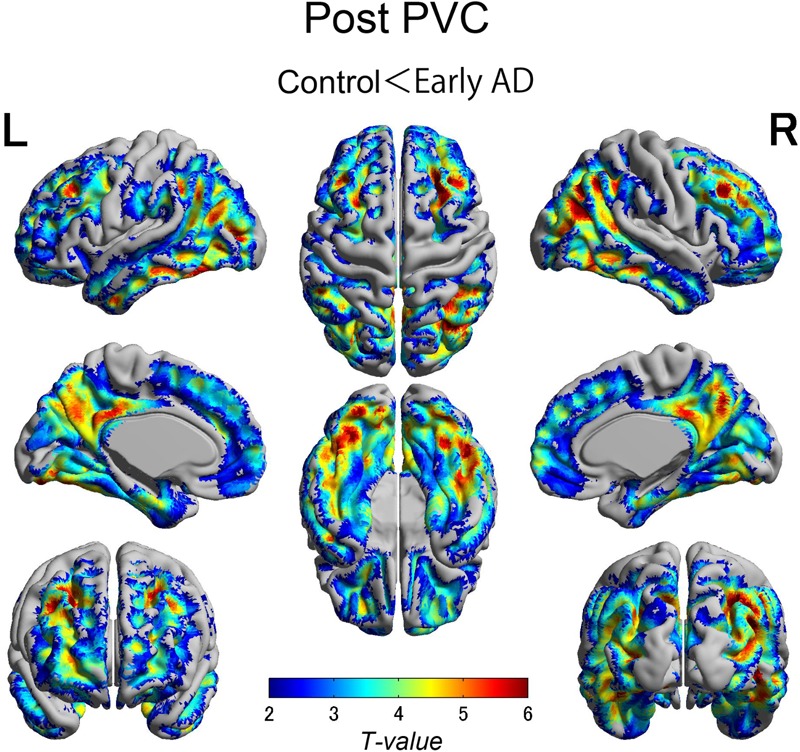
The distribution of voxel-wise group differences after PVC with ^18^F-THK5351. Almost the same tracer retention was observed as that before PVC. PVC, partial volume correction.

**FIGURE 3:**
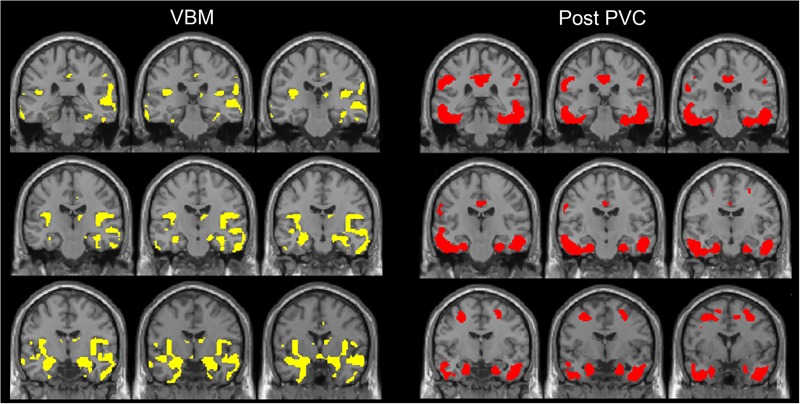
The distribution of voxel-wise group differences between early AD patients and controls. A series of coronal sections of VBM (yellow) and Post-PVC with ^18^F-THK5351 (red) along an anterior–posterior axis through hippocampus are mapped on a standard anatomical space.

The early AD patients showed significantly increased tau deposits on ^18^F-THK5351 PET data before and after PVC compared with the healthy controls (**Figures 1, 2, 3** and **Tables 2, 3**). We found widespread neocortical tracer retention predominantly in the posterior cingulate and precuneus areas, as well as in the inferior temporal lobes, inferior parietal lobes, frontal lobes, and occipital lobes; the primary sensory and motor regions were spared. No significant difference of tracer retention was found in the white matter or subcortical structures between the two groups. The pattern of tracer distribution was similar between before and after PVC. PVC marginally reduced the size of clusters throughout the neocortex and no new clusters were produced, suggesting that PVC had little effect.

**Table 2:** Clusters of increased tau accumulation with early AD patients compared to healthy controls before PVC.

Region volume (mm^3^)	*t*-Value	*Z*-Score	Talairach coordinates (x, y, z)	Cerebral region
8,852	7.71	6.23	36, -64, 3	Right middle occipital gyrus
435	6.55	5.54	-8, -78, -3	Left lingual gyrus
799	6.21	5.32	-55, -37, -7	Left middle temporal gyrus

*The results were threshold at *p* < 0.001 (voxel level) and FWE corrected to *p* < 0.05 at the extent level. The coordinates refer to the Talairach reference space.*

**Table 3:** Clusters of increased tau accumulation with early AD patients compared to healthy controls after PVC.

Region volume (mm^3^)	*t*-Value	*Z*-Score	Talairach coordinates (x, y, z)	Cerebral region
1,566	7.56	6.15	25, -48, 43	Right precuneus
502	6.87	5.74	30, 30, 32	Right middle frontal gyrus
530	6.76	5.67	48, -56, -2	Left inferior temporal gyrus

*The results were threshold at *p* < 0.001 (voxel level) and FWE corrected to *p* < 0.05 at the extent level. The coordinates refer to the Talairach reference space.*

**FIGURE 4:**
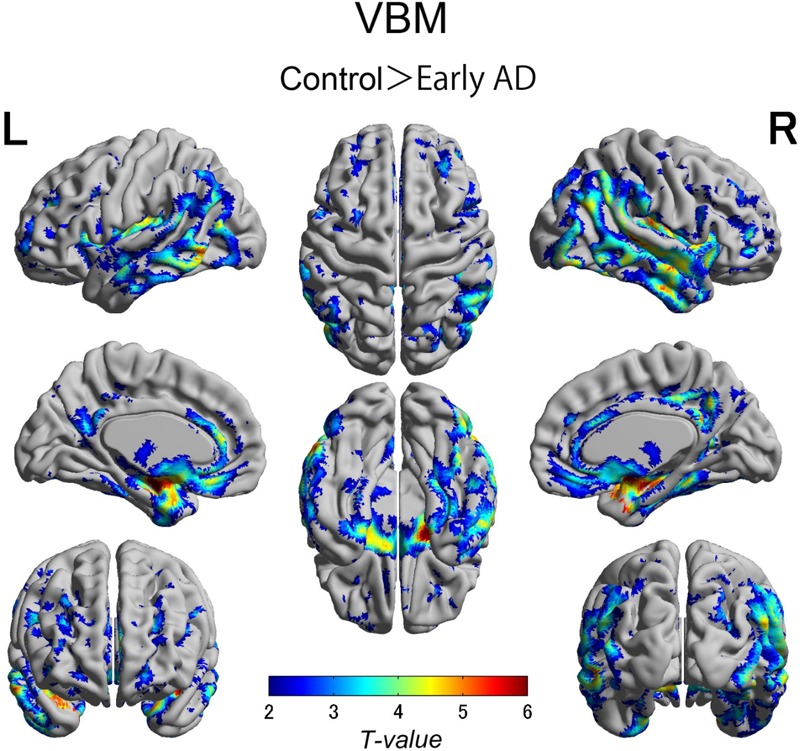
Voxel-wise group differences in gray matter volume between the early AD patients and healthy controls. Significant atrophy was observed in the medial temporal lobes including hippocampal head extending into amygdala and lateral temporal lobes as well as basal frontal lobes in the AD patients compared with the controls. AD, Alzheimer’s disease.

We also observed gray matter atrophy in the medial temporal lobes including hippocampal head extending into amygdala and lateral temporal lobes, as well as basal frontal lobes in AD patients (**Table 4** and **Figures 3, 4**). No significant difference of atrophy was found in the white matter or subcortical structures between the two groups.

**Table 4:** Clusters of gray matter loss with early AD patients compared to healthy controls.

Region volume (mm^3^)	*t*-Value	*Z*-Score	Talairach coordinates (x, y, z)	Cerebral region
10,885	7.02	5.83	-30, -7, 18	Left insula
446	5.53	4.86	4, -49, 26	Right cingulate gyrus
970	5.32	4.71	-57, -58, -2	Left middle temporal gyrus

*The results were threshold at *p* < 0.001 (voxel level) and FWE corrected to *p* < 0.05 at the extent level. The coordinates refer to the Talairach reference space.*

**FIGURE 5:**
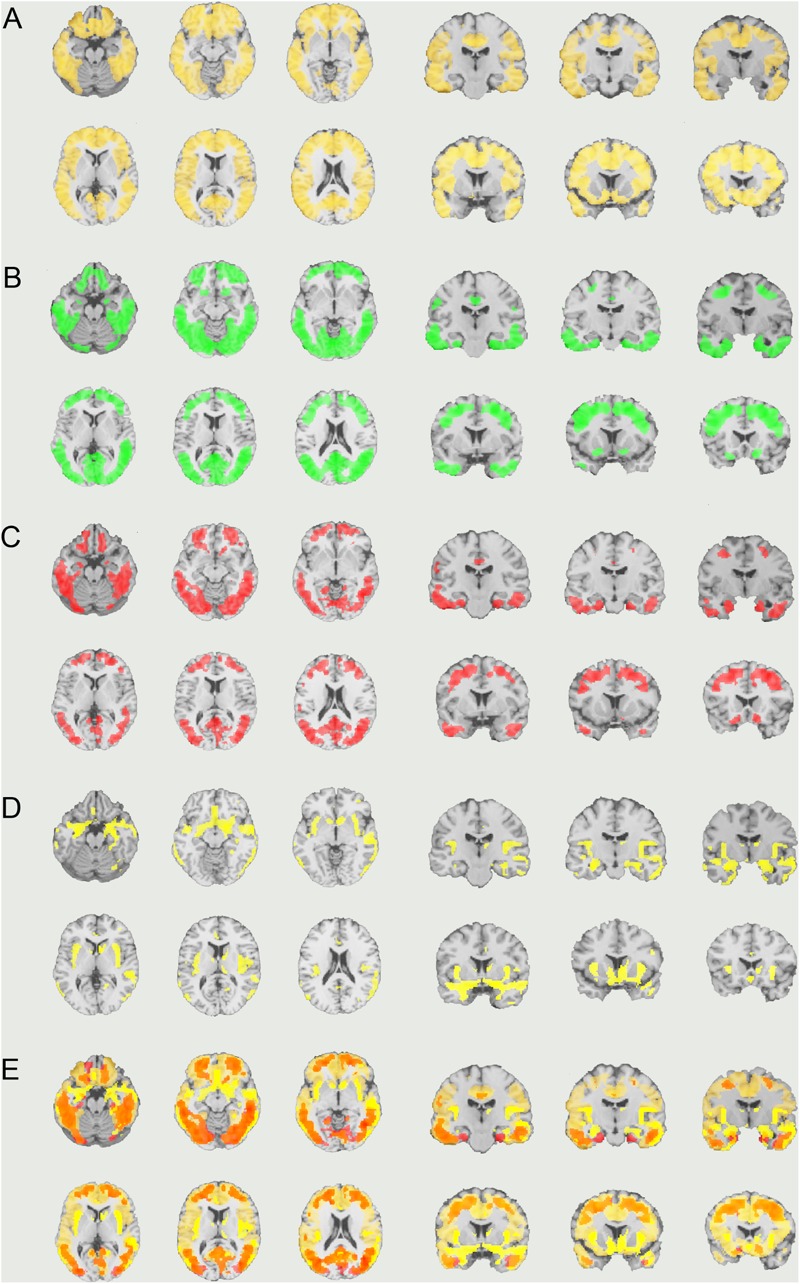
The distribution of voxel-wise group differences between early AD patients and controls. **(A)** PiB (bright yellow), **(B)** Pre-PVC with ^18^F-THK5351 (green), **(C)** Post-PVC with ^18^F-THK5351 (red), **(D)** VBM (yellow), and **(E)** PiB + Post-PVC with ^18^F-THK5351 + VBM results are mapped on a standard anatomical space. AD, Alzheimer’s disease; PVC, partial volume correction; VBM, voxel-based morphometry.

**FIGURE 6:**
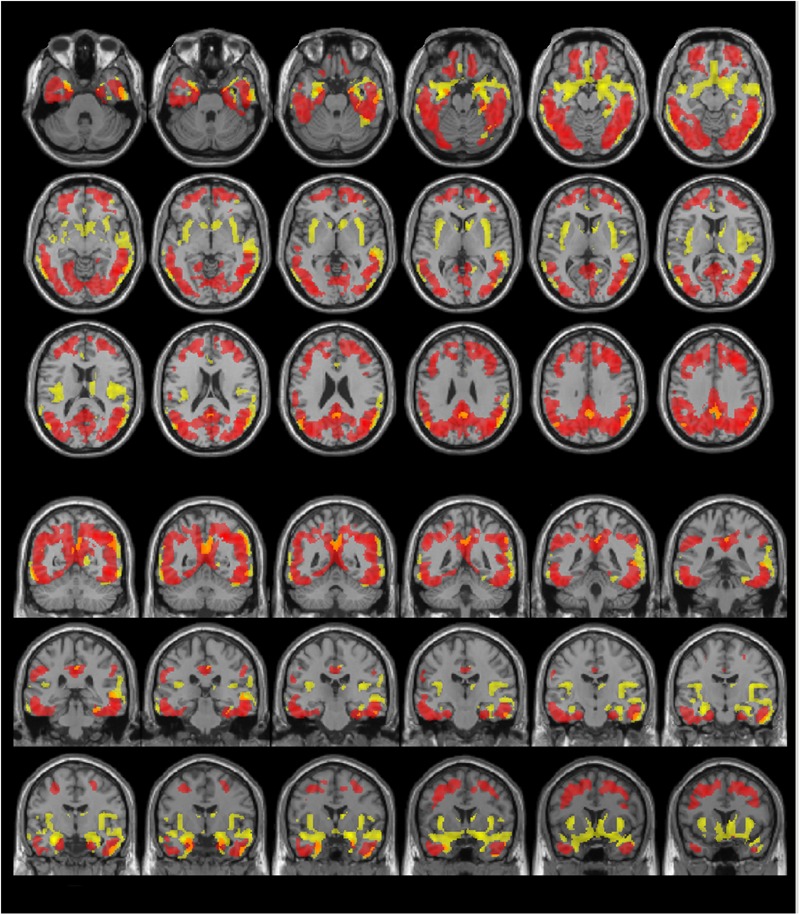
The distribution of voxel-wise group differences between early AD patients and controls. Post-PVC with ^18^F-THK5351 (red) and VBM (yellow) results are mapped on a standard anatomical space. Visual comparison of tau deposits after PVC with ^18^F-THK5351 and atrophy revealed only slight overlap in the precuneus, posterior cingulate gyrus, lateral parietal cortex, inferior/lateral temporal cortex and amygdala. AD, Alzheimer’s disease; PVC, partial volume correction; VBM, voxel-based morphometry.

Visual evaluation of the atrophied areas and tau deposits measured by ^18^F-THK5351 PET after PVC in AD patients as compared to healthy controls revealed only slight overlap in the precuneus, posterior cingulate gyrus, lateral parietal cortex, inferior/lateral temporal cortex, and amygdala (**Figures 5, 6**). Among them, only few areas showed significant correlations in direct comparison of gray matter volume and PVE-corrected ^18^F-THK5351 retention (**Table 5**). An FDR correction showed significant negative correlations in the right fusiform gyrus (*r* = -0.78, *p* = 0.00012), left fusiform gyrus (*r* = -0.65, *p* = 0.0036), left inferior temporal gyrus (*r* = -0.73, *p* = 0.00059), right lingual gyrus (*r* = -0.66, *p* = 0.0032), left lingual gyrus (*r* = -0.76, *p* = 0.00023), right middle temporal gyrus (*r* = -0.72, *p* = 0.00075), left middle temporal gyrus (*r* = -0.75, *p* = 0.00038), and left parahippocampal gyrus (*r* = -0.71, *p* = 0.00089) in AD patients. The right fusiform gyrus, left lingual gyrus, and left middle temporal gyrus correlations survived Bonferroni correction for multiple comparisons. No significant negative correlations were found in healthy controls in this direct comparison.

**Table 5:** Significant negative correlations between gray matter volume and ^18^F-THK5351 retention in early AD patients.

	Bonferroni correction	FDR correction
Right fusiform gyrus	*p* = 0.00012	*p* = 0.00012
Left fusiform gyrus	n.s.	*p* = 0.0036
Left inferior temporal gyrus	n.s.	*p* = 0.00059
Right lingual gyrus	n.s.	*p* = 0.0032
Left lingual gyrus	*p* = 0.00023	*p* = 0.00023
Right middle temporal gyrus	n.s.	*p* = 0.00075
Left middle temporal gyrus	*p* = 0.00038	*p* = 0.00038
Left parahippocampal gyrus	n.s.	*p* = 0.00089

*p-Values were Bonferroni and FDR corrected for multiple comparisons. n.s., no significant.*

A significant correlation with ^18^F-THK5351 retention and cognitive test scores (MMSE and CDR sum of boxes) within AD patients was not detected.

## Discussion

In this study, we compared tau deposits as measured by ^18^F-THK5351 PET before and after PVC along with regional brain atrophy using VBM. PVC was applied to correct for atrophy but had little effect, even in atrophied areas such as the medial/lateral temporal lobes and basal frontal lobes. Somewhat surprisingly, only a few associations were found between atrophy and tau deposits. The finding that tau load seemed to show very few relationships with gray matter atrophy has never been reported in previous tau PET studies.

A pathological hallmark of AD is that NFTs first form in the transentorhinal cortex, followed by the hippocampus, spread from the nearby cortex (predominantly the limbic, insular, and basal frontal areas) to the posterior cingulate cortex, and then spread throughout the neocortex (Braak and Braak, 1991; Braak et al., 2006, 2011). Previous tau PET studies reported tracer retention in the neocortical regions, most prominently throughout the temporal lobes, posterior cingulate/precuneus, and parieto-occipital and frontal lobes, whereas the primary sensory, motor, and visual areas were spared (Villemagne et al., 2014; Johnson et al., 2016; Ossenkoppele et al., 2016; Schöll et al., 2016; Maass et al., 2017). The widespread distribution of tau deposits in neocortical areas detected in our study was consistent with the results of previous studies (Serrano-Pozo et al., 2011; Villemagne et al., 2014; Johnson et al., 2016; Ossenkoppele et al., 2016; Schöll et al., 2016; Maass et al., 2017). Our results also corresponded to neuropathological findings in AD patients (Braak stages V/VI) (Braak et al., 2006, 2011).

We performed PVC to correct for brain atrophy. The pattern of tracer retention was similar between uncorrected and corrected data, suggesting that PVC had little effect, in line with previous studies with ^18^F-AV-1451 (Ossenkoppele et al., 2016; Schöll et al., 2016; Maass et al., 2017). A potential explanation for ineffectiveness of PVC was that we evaluated early AD patients with mild brain atrophy. However, VBM analysis revealed significant atrophy in the medial/lateral temporal lobes and basal frontal lobes in the AD patients. We could not detect significant tau deposits in these atrophied areas, even after PVC. This significant discrepancy between tau deposits and atrophy revealed in the present study might help to explain the mechanisms of ^18^F-THK5351 retention.

^18^F-THK5351 retention was detected in less atrophied areas in the neocortex but not in the most severely atrophied areas; these findings have not previously been reported. According to pathological studies, the spread of NFTs in the neocortex leads to synaptic dysfunction, glial activation, and neuronal loss (Sperling et al., 2014). At the neuronal cytoskeleton level, NFTs begin with the accumulation of soluble phosphorylated tau in cell bodies. As the phosphorylation progresses, this pretangle gradually aggregates and forms insoluble intracellular mature NFTs. After neuronal death, the abnormal hyperphosphorylated tau becomes exposed as extracellular ghost tangles (Braak et al., 1994, 2011). According to previous studies (Harada et al., 2016b; Ono et al., 2017; Saint-Aubert et al., 2017), all three families of radiotracers (^11^C-PBB3, ^18^F-THK5117, ^18^F-THK5351, and ^18^F-AV-1451) are insensitive to pretangles. However, ^11^C-PBB3, ^18^F-THK5117, and ^18^F-AV-1451 are sensitive to both intracellular mature tangles and extracellular ghost tangles. ^18^F-THK5351 is sensitive to tangles but discrimination between mature tangles and ghost tangles has not been demonstrated (Harada et al., 2016a). Lowe et al. (2016) recently reported that ^18^F-AV-1451 has a preference for mature tangles over pretangles and ghost tangles, suggesting that morphological differences might affect the binding intensity. If ^18^F-THK5351 has a similar preference, this could explain our results because areas with more severe neuronal loss have more ghost tangles (Fukutani et al., 1995), which might be less sensitive to tracer binding.

Interestingly, ^18^F-THK5351 retention was found in more advanced Braak stages compared with the Braak stage of atrophied areas revealed by VBM. A recent report on retrospective cortical atrophy and tau PET in clinically normal elderly adults revealed a correlation between retrospective parahippocampal atrophy and inferior temporal AV-1451 retention instead of parahippocampal retention, suggesting that tau pathology precedes atrophy (LaPoint et al., 2017). While our results support this hypothesis, longitudinal analysis of the association between tau pathology and atrophy are needed.

Although hippocampal atrophy is a key structural finding in AD (Matsuda, 2016), we did not find significant differences in hippocampal ^18^F-THK5351 retention between the AD patients and controls even after PVC. Johnson et al. (2016) reported that AV-1451 retention in the hippocampus was relatively low, which is consistent with our findings. They considered that off-target binding in the choroid plexus might affect retention in the hippocampus. However, there must be another reason because the THK series does not have off-target binding in the choroid plexus (Ishiki et al., 2015; Harada et al., 2016a; Saint-Aubert et al., 2017). Schöll et al. (2016) reported that PVC produced only a small increase in tracer retention in the hippocampus, which is in line with our results. Recent imaging and pathological studies revealed that medial temporal atrophy and NFTs that are restricted to the medial temporal lobe in the absence of amyloid plaques are generally observed in cognitively normal healthy elderly adults (primary age-related tauopathy) (Crary et al., 2014; Jack et al., 2016; Shimada et al., 2016). Cognitively impaired individuals have a large proportion of ghost tangles in the medial temporal lobe compared with healthy elderly adults (Santa-Maria et al., 2012; Crary et al., 2014). Thus, we believe that individuals with mature tau deposits and patients with mild cognitive impairment or AD with increased tau deposits that are insensitive to tracer binding show small differences in tracer uptake in the hippocampus.

This study has several limitations. First, this study had small sample sizes of both early AD patients and healthy controls. Second, we did not collect genetic data (apolipoprotein E). Therefore, further longitudinal studies are needed to investigate whether tau deposits predict atrophy. Third, a recent human blocking study using monoamine oxidase B (MAO-B) inhibitor demonstrated significant reduction of ^18^F-THK5351 SUVs in cortical regions (Ng et al., 2017). Even the reference region such as cerebellar cortex, which is considered the least-affected region, also reduced ^18^F-THK5351 SUVs by 41.6%. Because ^18^F-THK5351 retention seems to reflect reactive astrocytes in addition to tau pathology, it is hard to reveal the exact correlation of atrophy and tau pathology. Further studies are needed to clarify ^18^F-THK5351 binding sensitivity to tau pathology and MAO-B.

## Conclusion

We applied PVC to correct for atrophy in early AD. However, the pattern of ^18^F-THK5351 tracer retention was similar between before and after PVC, suggesting that PVC is not required to estimate tau deposits. Moreover, ^18^F-THK5351 retention was detected in less atrophied areas but not in the most severely atrophied areas even after PVC. Although this discrepancy between tau deposits and atrophy could be ^18^F-THK5351 specific findings, our findings suggest that tau load precedes atrophy. Further prospective longitudinal studies of the association between tau pathology and atrophy are needed to confirm our findings.

## Author Contributions

YS, DS, and HM designed the experiments. HT, YY, MS, and TT collected and sorted the data. YS, DS, EI, NM, MO, and KK carried out the experiments, data analysis, and statistical analysis. YS, DS, and HM wrote the manuscript. SF, YK, NS, and NO revised the manuscript.

## Conflict of Interest Statement

The authors declare that the research was conducted in the absence of any commercial or financial relationships that could be construed as a potential conflict of interest.

## Footnotes

1 http://www.neuromorphometrics.com/
